# Waving through a window: Agricultural research faculty perspectives on science communication challenges

**DOI:** 10.1371/journal.pone.0304793

**Published:** 2024-06-14

**Authors:** Jamie Alexander Greig, Shelli Rampold, Emily Paskewitz, Taylor Ruth

**Affiliations:** 1 Agricultural Leadership, Education and Communications, The University of Tennessee, Knoxville, Tennessee, United States of America; 2 The Department of Communication Studies, The University of Tennessee, Knoxville, Tennessee, United States of America; Universidade do Sul de Santa Catarina, BRAZIL

## Abstract

This qualitative study explores agriculture research faculty’s challenges with participating in Science Communication. To explore the challenges shared by faculty, we utilized the proposed Faculty Science Communication Engagement Framework, which identifies three dimensions that may cause challenges for faculty Science Communication work: personal, professional, and institutional. During interviews with 11 research faculty, we identified Science Communication challenges within these dimensions. Participant challenges within the personal dimension include allocation of time, the learning curve, audience familiarity, and mass-media concern. Professional dimension challenges were “it’s not my job” and disciplinary norms, while challenges within the institutional dimension included a lack of support and resources. Across these dimensions, faculty challenges revolved around the time required to invest in Science Communication activities, the needed resources (personnel, technology, and financial), the value placed on efforts by their academic institution, and the lack of knowledge regarding Science Communication techniques and audience. These findings are described through rich data, and practical recommendations are provided for fostering future Science Communication engagement and interest among faculty. These include Science Communication training focused on specific content areas, hands-on training and support with Science Communication technologies, including Extension and non-Extension faculty in training sessions, creating structured and strategically implemented shared Science Communication resources at the institutional level, including Science Communication efforts in university strategic planning, and awarding and recognizing faculty who utilize Science Communication successfully.

## Introduction

Public engagement with science is a topic that has gained considerable attention in research, funding, and policy agendas in recent years. The frequent and rapid spread of misinformation online and through the media has led scholars to address science literacy among public audiences [1], which refers to how individuals understand, appreciate, and apply science in their day-to-day lives [2]. Members of the public often have to make decisions that require a basic understanding of science to be well-informed, yet many find it difficult to navigate and understand the intricate scientific information associated with these sources [1]. The National Academies of Science, Engineering, and Medicine [3] have emphasized the need for scientists to improve and increase their science communication efforts. This is to ensure that the public not only understands complex scientific topics but can also relate them to their own values and beliefs. However, NASEM also acknowledged science communication is a complex task and learned skill that invites an array of challenges in effective implementation. Considering the unique challenges associated with the daily intersection of sciences and public consumer audiences, it is imperative science communication efforts be encouraged and supported among research professionals [4].

Recent scholarship underscores that mere knowledge proliferation, as posited by the deficit model prevalent in the 1960s, is an insufficient framework for today’s intricate information landscape [5]. The rapid proliferation of misinformation in digital media necessitates a more nuanced approach to science literacy. Contemporary audiences must navigate a complex array of scientific narratives, requiring not only understanding but also the ability to critically evaluate and apply scientific concepts within diverse contexts [3]. As such, researchers face the challenge of not only conveying complex information but also contextualizing it within the values and belief systems of varied audiences.

Scientists are frequently considered trustworthy by the public in comparison to other figures like journalists, religious leaders, politicians, and business leaders [6], and their active engagement in public science communication could play a significant role in shaping the public’s confidence in science and their understanding of scientific concepts. However, scientists have historically received limited training in science communication, making this task difficult to accomplish [7, 8]. For example, while graduate educators recognize the importance of science communication, there is a need for more training opportunities and better integration of communication skills into curricula [9, 10]. Yet, when scientists do engage in Science Communication, they have improved their communication skills, increased audience enthusiasm and knowledge, and contributed to civic education by defending science from misinformation, educating the public, and building trust [11–13]. Due to these benefits, an increased number of sectors are calling for Science Communication activities [14].

In agriculture and natural resources (ANR), Science Communication is critical to ensuring cutting-edge technology, innovations, and recommended behaviors that benefit our environment and food supplies reach targeted end users [15, 16]. Engaging members of the public who impact and are impacted by ANR activities can help minimize the spread of scientific misinformation and enhance the potential positive impact of scientists’ work [17]. Science communication can improve public acceptance and trust in science, but its effectiveness may depend on the audience’s existing trust in science and the communication’s focus on addressing misinformation and educating the public [18, 19]. However, public trust in science has had a noticeable decline throughout the lifetime of the COVID-19 pandemic [6].

Science Communication is a complex, multifaceted endeavor, and faculty members may face numerous challenges to effectively engage in such activities [12]. Prior research supports the hypothesis that faculty members are unlikely to pursue Science Communication if they consistently face challenges that impede their abilities to do so effectively [8, 14]. In the context of Science Communication, a *challenge* can be defined as “a major and complex barrier to effective communication that, while difficult to overcome, could be addressed by filling current gaps in knowledge about the nature of the challenge and how it can be overcome” [3]. Identifying the gaps in knowledge related to Science Communication challenges, as well as attitudes toward public engagement [18], can provide a road map to direct future research and training and ultimately enhance the quality and effectiveness of Science Communication. Therefore, we sought to understand the unique challenges experienced by agriculture research faculty members that hindered their engagement in Science Communication. Research of this nature can provide practical recommendations for encouraging and supporting Science Communication endeavors among these faculty types.

An element that distinguishes ANR research from other fields is engagement with the public, typically via state extension services. U.S. Land-grant universities, established under the Morrill Act of 1862, were designed to democratize education and ensure the practical application of scientific knowledge, especially in agriculture [20]. Through agricultural experiment stations and cooperative extension services, Land-grant generated agricultural research has a dirct connection with the public [21]. The land-grant system, with its emphasis on "usable science," presents a unique case where scientific research is intrinsically linked to direct communication with the public [20]. Unlike other scientific disciplines, where the dissemination of research may be limited to academic publishing or peer-to-peer communication, agricultural research at land-grant institutions is legislatively tasked with extending beyond the laboratory and into the fields of farmers and agricultural businesses. The cooperative extension services act as a critical boundary organization that not only disseminates information but also facilitates a two-way dialogue [22]. In addition to this legislated mandate, faculty at land-grant universities often have split-appointments. Faculty roles can be assigned research, teaching, or extension responsibilities. While some have one area of responsibility others may be split. This creates a unique dynamic among this research population where some faculty may have more science communication emphasis placed on their role than others.

Recognizing the unique challenges faced by agricultural researchers in communicating science effectively, this paper explores the specific barriers and offers insights into how they might be mitigated. The aim is to identify knowledge gaps and attitudinal nuances among researchers that, once addressed, could significantly enhance the caliber and impact of Science Communication efforts in the field of agriculture and natural resources.

## Conceptual framework

**Fig 1:**
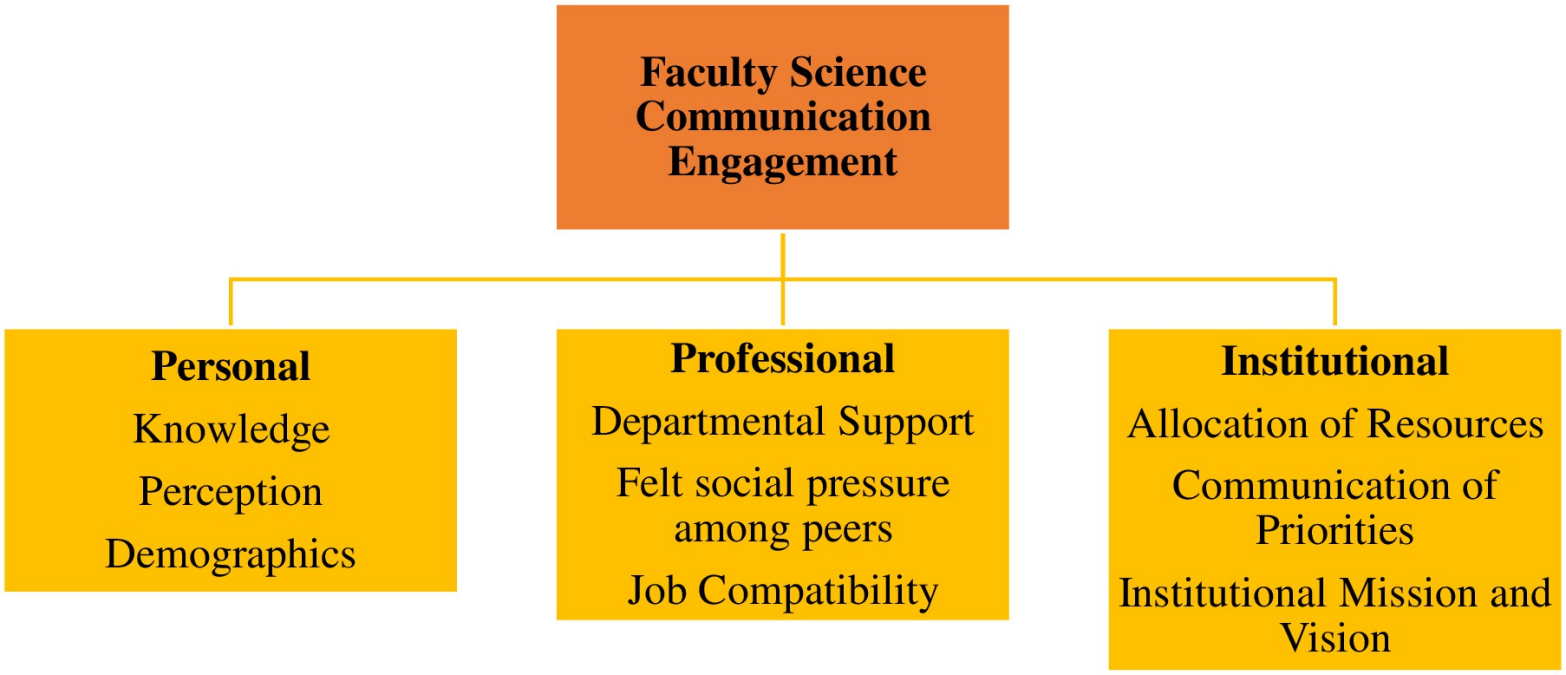
Faculty science communication engagement framework.

To guide our analysis and discussion of the results pertaining to faculty members’ unique challenges, we adapted a conceptual framework (Fig 1) originally developed to explore faculty members’ engagement in study abroad [23]. Similarly to study abroad, within academia science communication is often framed as additional voluntary work. This means it is not described as something that is inherent to the function of a researcher but rather are self-selected activities. While at first the model may seem simplistic, this framework was chosen as it provides a practical structure with which faculty insights can be referenced. The ultimate goal of this research is to provide recommendations to the field. By allocating “challenges” to the personal, professional, and institutional dimensions, we identify practical pathways for these recommendations.

The original framework included three dimensions that contained factors hypothesized to influence faculty members’ study-abroad engagement. For this study, we identified new sets of factors within each dimension hypothesized to influence faculty members’ engagement in Science Communication activities. Factors were selected for inclusion in each dimension based on an extensive review of prior science communication literature. Per the current proposed framework, faculty members’ engagement in Science Communication is influenced by factors present at the personal, professional, and institutional dimensions. We applied this organizational framework to specifically discuss the challenges faculty members face and what those challenges are at each dimension (i.e., personal, professional, and institutional). For instance, ’perceived pressure,’ a variable traditionally associated with professional obligations, is included to reflect the complex social dynamics within the professional sphere that may motivate or deter engagement in Science Communication. Such pressures may be explicit, like departmental expectations, or implicit, such as perceived reputational impacts. While we acknowledge that there can be intersectionality between dimensions, this specificity in categorization enables a targeted exploration of faculty experiences and attitudes towards Science Communication. In our adapted framework, we underscore the dynamic interplay between the three dimensions, recognizing that challenges often do not arise in isolation. For example, a lack of personal value placed on Science Communication may be compounded by a professional environment that does not offer recognition or support for these activities [24]. This intersectionality is crucial for understanding the compound effects of barriers across different spheres. Additionally, this framework provided a means of organizing the analyses and discussion, and, in turn, the results of this study provided further insight into unique characteristics to be included and explored within each dimension of the framework.

### Personal dimension

Personal dimension challenges are those that are unique to the individual faculty member. Such challenges may include lack of perceived value or importance of communicating science to public audiences, gaps in knowledge or skills in how to communicate their science, lack of time, or other factors. In prior research, scientists were less likely to engage in science communication if they did not perceive themselves as knowledgeable about methods of Science Communication [25, 26]. Similarly, factors such as negative attitudes toward communicating science to the public have posed challenges to faculty members’ engagement in Science Communication activities [16]. Besley [27] determined scientists’ dedication to public service and perceived self-efficacy related to science communication were strongest predictors for public engagement, and Dudo [7] also concluded there was a strong, positive relationship between scientists’ attitude toward science communication and their engagement in these activities. These positive perceptions toward Science Communication have been seen more predominately across younger generations of scientists [14]. This body of literature establishes personal dimensions, such as attitude, as key antecedents to Science Communication.

### Professional dimension

The professional context can also influence faculty members’ engagement in Science Communication. In this study, we consider the professional dimension factors as those within faculty members immediate professional environment, such as support of department heads or advisors, felt social norms of communicating science within the department or professional field [28, 29], or natural fit or compatibility of Science Communication with their scientific content [30]. Many faculty do not believe their peers recognize the need for Science Communication or find the time to engage in these types of activities [31]. Some faculty have even reported receiving criticism from other scientists when attempting to engage in Science Communication [25]. However, researchers have concluded scientists will continue to engage in Science Communication despite not feeling supported by their peers or administrators [14, 26]. Yet other professional dimensions, like appointment type and science content area have been found to decrease engagement in Science Communication [26, 32]. Additionally, faculty have historically had to prioritize publishing and securing grant funding over Science Communication due to lack of professional incentives and time for the latter [33].

### Institutional dimension

While the professional dimension characteristics speak to the nature of a faculty members’ professional field or direct department, institutional dimension characteristics pertain to those of the larger institutional structure [34]. For this study, the institutional dimension was used to categorize the [University] challenges at this level that can impede a faculty members’ successful engagement in Science Communication activities. For example, institutional support of faculty efforts is a significant factor in determining whether faculty members would engage in a particular activity, which has resulted in a call for increased institutional support, resources, and recognition for engagement in Science Communication [33, 35].

### Research questions

The purpose of this study was to identify and describe the unique influences experienced by agriculture research faculty members that hindered their engagement in Science Communication and provide practical recommendations for supporting Science Communication endeavors among research faculty.

The following research questions (RQ) guided this study:

RQ 1: How do [University] AFNR faculty members’ perceived personal dimensions create barriers for engaging in Science Communication.
RQ 2: How do [University] AFNR faculty members’ perceived professional dimensions create barriers for engaging in Science Communication.
RQ 3: How do [University] AFNR faculty members’ perceived institutional dimensions create barriers for engaging in Science Communication.

## Methods and procedures

The current study is a component of a broader project investigating perceptions of Science Communication at [University]. The project commenced with a quantitative evaluation of faculty members’ experiences, perceptions, and barriers related to Science Communication. The present study details the qualitative phase, which encompasses follow-up interviews that provide rich contextual data on this topic.

### Sample

After obtaining approval from the Institutional Review Board, we extended invitations for semi-structured interviews to all faculty members at [University] with formal research appointments (totaling 205). These faculty members included those with combined responsibilities in research, extension programming, and/or teaching. Initial contact was made via two informational emails sent by the Research Dean’s Office over a two-week period, introducing the study and soliciting participation in an initial survey. From the survey respondents, individuals who expressed interest in participating in further discussions (n = 20) were contacted by the research team to schedule an interview. A subsequent email secured interview commitments from 11 faculty members.

Table 1 presents the appointment information for these interviewees, including their area of focus, appointment type, and prior involvement with Science Communication. Interviews were carried out from August 15 to August 23, 2022, with durations ranging from 20 to 52 minutes (average = 31 minutes). The interviews were recorded and transcribed via the Zoom platform, with transcription accuracy verified by the interviewing author. This process yielded 176 pages of transcript data (average = 16 pages per interview).

**Table 1:** Participant characteristics from interview data.

	Content area	Primary role	Experiences with public communication
Participant 1	Plant Sciences	Teaching	Local clubs, homeowner workshops, schools
Participant 2	Livestock	Extension	Extension articles and presentations, commodity federations, international producers
Participant 3	Microbiology	Research	K-12 teachers, youth through extension and 4-H
Participant 4	Livestock	Extension	Producers and Tennessee stakeholders, social media, extension pubs and factsheets
Participant 5	Agricultural Economics	Research	State government, local news
Participant 6	Plant Sciences	Extension	Homeowner, commercial, and consumer group presentations, contact for extension agents
Participant 7	Agricultural Economics	Research	Department staff written press releases, local news
Participant 8	Natural Resources	Administration	Programming at research station
Participant 9	Livestock	Administration	Trainings with extension agents
Participant 10	Agricultural Economics	Extension	State and local government
Participant 11	Food Science	Research	Peer reviewed articles, conference presentations

*Note*: Experiences with public communication are how each participant defined their involvement with public communication activities.

### Procedure

Interview questions were created in consultation between three authors. The interview questions were developed based on prior research pertaining to the science communication experiences of faculty members across various fields. This review helped identify recurring themes and challenges related to Science Communication, which in turn guided the structure and content of the interview protocol. Specifically, the interview questions sought to identify the nature of faculty members’ research, their experiences in communicating this research to the public, challenges faced, strategies adopted for overcoming these challenges, perceptions of professional and institutional support, and training needs in the realm of Science Communication. The aim was to capture a holistic view of the myriad factors that might influence faculty members’ engagement with Science Communication in the agricultural research domain.

A concept driven coding process was adopted for analysis. Concept driven coding is useful when researchers have a sense of what can be found in the transcripts based on previous studies, topics in the interview guide, or based on the literature review [36]. Researchers first create a set of codes based on this information and some of the transcripts, and then code the data. During the coding process, original codes may change or evolve based on emergent ideas from the interviews [37]. Concept driven coding allows researchers to focus analyses on theoretical ideas of interest in the interviews, and then identify themes within those areas from the data.

To ensure credibility and reliability in data, three key procedures were used while coding: triangulation, creating an audit trail, and utilizing a codebook. First, the coders relied on a balance between individual coding and group discussion to clarify codes and project foci. This balance between individual and group coding is a form of triangulation where multiple researchers can help reduce bias in the results [38]. During group discussions, the work of others was checked for agreement and held any discussion before proceeding to the next stage of individual coding. Second, the coders engaged in individual and group level memoing maintain an audit trail for our coding procedures and changes [36]. These memos were used during discussion to ensure each author’s perspective was shared given the different interpretations possible from the data [38].

To begin coding, all 11 transcripts were individually reviewed for common themes and ideas around perceptions of science communication. Each author individually engaged in memoing, then met to discuss memos and determine key codes. After meeting to discuss initial areas of interest, the first author created a codebook for further coding. A codebook serves as a guide to coding for researchers and can help increase reliability in qualitative coding [39]. The codebook included definitions of codes and exemplars and dimensions of all codes. This codebook consisted of six areas based on prior research, the interview guide, and the initial read of the transcripts. These codes included: personal Science Communication challenges, personally conducted activities, personal Science Communication characteristics, others’ Science Communication challenges, others’ conducted activities, and others’ Science Communication characteristics. The first author coded two transcripts with this codebook, then sent the codebook and transcripts to another author for a coding check.

After the initial round of coding, the authors reviewed and discussed the codes to refine their definitions and illustrative examples. It was determined that the codes needed revision due to overlaps among the six original categories. Consequently, the codebook was refined, allocating codes to the three dimensions identified in the theoretical framework: personal, professional, and institutional. In the second round of coding, excerpts from the initial coding process—specifically those tagged as personal challenges and others’ challenges—were reclassified into the aforementioned three dimensions. The categories were examined for coherence, and it was agreed that the three dimensions corresponded well with the data. Subsequently, two of the researchers developed themes within the personal, professional, and institutional dimensions to pinpoint specific challenges that research faculty encounter when engaging with Science Communication.

The researchers also recognize their potential research biases as faculty members at [University], which may have influenced participation and the interpretation of the results. Moreover, considering that the invitation to partake in the project was issued by the Research Dean’s Office, there is a possibility that participants might have felt compelled to join, potentially influencing the outcomes.

## Findings

### RQ 1

*Time and the learning curve*, *audience familiarity*, and *mass media concern* were common barriers to Science Communication when discussing participants’ personal dimension barriers to Science Communication. While allocation-of-time concerns were common throughout the interviews, we noted that the time challenge was often linked to the learning curve associated with Science Communication. Many participants described the challenge of allocating time to activities requiring them to learn additional communication skills. In addition, participants expressed a challenge of audience familiarity, including how to deliver messages to a public audience and mass-media concerns around how the media interprets and presents their research.

#### Time and the learning curve

For many of our participants, allocating time represented a significant challenge for engaging in Science Communication. One participant (6) noted, “Unfortunately, there’s only so many hours in a day…I just need more time”, while another (3) stated, “I struggled to set aside time to say ‘Okay, this is important enough for me right now to go do that.’” In addition, many participants did not have experience or training in Science Communication, making allocating time to these efforts challenging or unattractive. One participant (1) said:

There’s a pretty high learning curve, so I’m not going to do it. Whereas if I was exposed to some ways to really easily create content and I was given the step-by-step kind of recipe…this is how you do it, and then you can start learning and, as you get better you’ll iterate and get better and better and better at it. But this is a way that you can create really efficiently some high level…high-quality content…then it’s something that I’d be more willing to consider including in some of the things that I do.

Another participant (11) shared, “I don’t know much and I’m not willing to spend much time to learn on my own. If someone is giving me some hints, and I will workshop, I’m willing to learn, but I’m not going to explore this myself.”

More specifically, some participants talked about the technology learning curve as prohibitive to Science Communication efforts. Participants linked Science Communication with technology and social media, and explained how their lack of comfort with technology makes the effort challenging. Interestingly, participants described this lack of comfort from the perspective of personal identities. Some participants identified technology-use for Science Communication as a generational identity concern:

“I think, it’s just also beyond the older generation. I feel like I’m almost at the cusp of it, where I’m like ‘oh my gosh what is TikTok.’” (Participant 4), “I know I’m a kid of the Internet age, but I’m not great at social media or posting.”

(Participant 6)

One participant (9) noted that their preference for face-to-face, one-on-one conversations makes Science Communication efforts challenging because technology is so crucial to all those efforts. They concluded,

“I’m more comfortable with a one-on-one setting than I am [with] all of this technology, because I’m really not a good technology guy”, later noting they are “not a good blogger”. In addition, participants noted the characteristics of various technologies presented a challenge to engaging in Science Communication. Many participants struggled with knowing how to utilize suggested technologies to engage in Science Communication. For example, one participant (1) discussed the learning curve with using multimedia equipment:

I’ve got to figure out what I’m going to use it (multimedia equipment) for and then, after I’ve done all of that, then I have to actually go out and use it to capture what it is I’m going to use it for and…that’s a pretty big ask to add on to all the other stuff that I’m doing on a regular basis, and so a lot of times… I might use it for one or two things and go ‘yeah, okay, I’m good.’

Participants noted the need to learn new technology systems presented a major learning curve to making Science Communication efforts happen. Other participants explained how their unfamiliarity with social media made Science Communication challenging, with one stating (11), “which one [social media site] is a credible?”. Another participant (4) expressed a concern with the time required to post frequently enough on social media to ensure, “it’s actually popping up on other people’s notifications.”

Finally, some participants struggled to prioritize Science Communication when they knew a learning curve was present. For example, some participants questioned the importance of Science Communication efforts when compared with tasks that they identified as being more necessary for their position. One participant (3) shared, “I would sit down and look at (learning a new media technique) and think about whether my time is more valuable…learning how to put together a video…the mechanics of it, or can I put that time to developing some new curriculum content for my teachers.” This participant struggled to find a way to incorporate Science Communication efforts, with the associated learning curve, alongside their other research faculty duties. In another interview, a participant (1) stated,

I don’t necessarily have the bandwidth to spend my time figuring out, from a director’s perspective, how I’m going to put all that together, and then create some end product that’s going to be, you know, 10-or-15 minutes’ worth of content. It just becomes cost prohibitive in terms of time.

#### Audience familiarity

In addition to the time and learning curve challenge, participants also discussed issues in understanding and connecting to the Science Communication audience. Some participants discussed their struggle in connecting their research with the public. One participant (3) noted, “I remember that…something that I was particularly challenged by was how do I convince people that an animal decomposing in the woods is important for the food on their table.” For another participant (9), their challenge was translating their scientific understanding of the process in a way the “general public can understand”. A different participant (2) with an extension appointment had the opposite challenge, noting that they struggled delivering information to a research-specific audience but had no problem, “talking to someone in a cow pasture or a chicken house.”

#### Mass-media concern

Participants noted the challenge of communicating with the public via media outlets since messages can be misrepresented or misinterpreted. For example, one participant (3) shared:

One of the things I always struggle with is that with as scientists we’re so detailed and nuanced in what we what we do and say, and the media really likes a good sound bite…oversimplifies things and it washes over a whole bunch of that nuance that we as scientists think is really, really important and… that’s always just going to be a challenge …but how to get to those simple distilled messages without completely washing over the meaning and I’ve certainly had cases where… a reporters interviewed me, and you know we’ve talked to them… and I just felt like things were really way over simplified. And I know that’s part of packaging it for consumption by a general public readership. But it can be a little bit frustrating to see something that you know to be really complex and nuanced just sort of boil down to a single sentence.

A few participants noted they were concerned if they would say the right thing, especially on film or in voice recording, with some also noting that Science Communication efforts were dangerous, especially when reputations are in jeopardy. One participant (11) shared:

The past couple times (faculty invited) to go talk on radio…about certain things. People tend to reject those. We don’t want to do this if you say a couple things wrong or they can just cut to just take this portion…and they can make up a story whatever way they want so your reputation is in jeopardy…so public communication is…it can be dangerous and we don’t know how…it can be time consuming or dangerous. I don’t know why people want to do this. I know it is important.

The risk to career was brought up by participants as a cautionary tale to engaging in Science Communication. For these faculty, they felt it was “better to be safe” [7] and not engage in Science Communication rather than risk their career and research over a media statement taken out of context.

### RQ 2

Turning to professional dimensions, faculty also noted ways their departments, colleagues, and fields presented challenges to Science Communication efforts. These challenges focused more on the broader issues tied to job duties and responsibilities, and the need to choose between perceived required job duties and nonessential tasks. The themes noted in this area include: *It’s Not My Job* and *Disciplinary Norms*.

#### It’s not my job

For many of the participants, Science Communication efforts were seen as something outside of their job responsibilities. This came from many different avenues, including job appointments and stage in the career. Most participants associated Science Communication efforts with faculty who have an extension appointment. The link between extension appointments and Science Communication efforts was explained primarily by job duties. For example, some participants noted extension appointment faculty are the ones who really need to worry about Science Communication with one participant (1) stating, “Really, unless we have an extension appointment, we are not evaluated based on how much we talked to the public at all.” Another (7) explained, “I do very little public-facing because I don’t have an extension appointment.” For these participants, extension appointment faculty were tasked with Science Communication, and it was not a requirement of other faculty positions.

Tied directly to this issue is the lack of value placed on Science Communication for faculty. Numerous participants noted their departments did not place value on Science Communication for non-extension faculty. One participant (5) summarized it by stating, “I would say, you know it’s valued in extension for that’s also part of their job.” The “not my job” idea was common for faculty, as many turned to their evaluation materials as evidence of what tasks they needed to focus on. A participant (4) noted: “I’ll invest my time in those activities if I believe that it will help keep my job.” Another (7) stated:

What I’m evaluated on is my number of presentations, number of publications, and dollars of grant funding I bring in… there’s nowhere that I actually evaluate it or required to for my performance ratings or for tenure and promotion to do anything with the public.

So, I don’t actually spend time doing that.

Faculty clearly placed Science Communication efforts in the hands of extension, and rarely saw it as part of their job requirements outside of an extension appointment. This likely comes from the lack of department evaluation of Science Communication efforts in performance reviews. As one participant (1) said, “It’s almost not even paid attention to.”

#### Disciplinary norms

During the interviews, participants noted some individuals struggle with Science Communication due to the norms of their academic discipline. One participant (4) summarized this by stating, “Someone, you know, working on a molecular problem in a lab. Trying to convey that to the general public, or the layperson is a very challenging thing. But a lot of them don’t have any skills or training in that.” Another participant (1) noted, “There’s a lot of fields that don’t have any interaction (with the general public).” The disciplinary norms for Science Communication were often used as justification for why some faculty do not participate in Science Communication efforts. One participant (3) noted graduate student training lacks an emphasis on the inevitable future Science Communication efforts required for tenure-track jobs.

They shared:

I’ve actually thought about Science Communication, a lot with the graduate students and postdocs that I work with. Especially as I see some of them come up through and have spent so much time in the lab and really don’t have a lot of exposure to how to communicate their science and they’re about to go out into the big wide world and they’re going to get calls from journalists and they’re going to have to go do that public engagement talk.

Disciplinary norms also played a role in how faculty described approaching Science Communication efforts, especially when looking at traditional academic education. A few participants noted academics are trained to communicate in very scientific ways that do not always translate to general public audiences. Participant 6 stated, “As academics we’re trained to do…the broad stuff…your methods and your statistics and your analysis and that doesn’t always go over well…sometimes that’s not the most effective way to tell the story.” Another participant shared:

I think that as scientists, the more that we can call upon those people (communication experts) to help us tell the story, because scientific writing is so boring I mean it is really dry…as scientists, some of us just aren’t educated on…how to communicate…to the public…we just talk to each other.

(Participant 5)

However, a few participants noted the challenges with Science Communication training efforts from an extension perspective:

Extension people…Most folks don’t realize, but they have a very difficult job because they’ve got to… sell this product to a bunch of different personalities and every person out there has a different mindset and has to be approached in a different way you can’t use the same techniques that you use with this person that you use with the person five miles down the road.

(Participant 2)

Participant 10 shared a similar sentiment:

Communicating science is not the easiest thing to do and extension folks, I don’t care whether they’re specialists like I am or whether they’re county agents…most folks might not understand it, but they have a difficult job in trying to do what I just said…Take scientific base knowledge and sell it to someone that basically knows nothing about this and convince them that there are advantages to you and to your operation. To try to change and do it in a different way or try to try something new, because…people don’t like to change.

### RQ 3

Participants noted ways the institutional structure, values, and resources of the university impacted Science Communication efforts. These challenges highlighted how the university itself constrains Science Communication efforts. These challenges are summarized as a *Lack of Support* and a *Lack of Resources*.

#### Lack of support

Many participants noted a lack of support from the university towards Science Communication efforts. Participants noted that the university does not include Science Communication efforts in evaluation or promotion reviews. One participant (1) stated:

I don’t think it’s valued in the sense that, when it comes down to … progressing as a faculty member, it’s kind of a nice thing to have but it’s not something that you have to do. And it’s not something that’s expected necessarily of what you do.

Another participant (9) brought up the tenure and promotion process, noting the system places emphasis on scientific communication, “via peer-reviewed research and then fundraising.” These quotes point to a lack of university support for engaging in Science Communication, especially for faculty without an extension appointment.

Another university level challenge was the perception that the university was focused on attention grabbing research. One participant (5) stated, “I feel like … anytime you want to…from the University’s perspective…relay your findings… you want them to be kind of groundbreaking and flashy to get attention.” This focus on potential bias in university communication efforts was a common idea from participants, with many also seeing a lack of emphasis by university officials on the extension efforts of faculty. One participant (4) shared:

On our side of campus we have [Institution] news and updates and notes and things like that. A lot of those updates and notes, and I’m not saying that those people observed shouldn’t be recognized, a lot of it seems to focus on Research and [College of Agriculture] initiatives and not a lot of it seems to trail over into the extension world.

#### Lack of resources

Finally, some participants also noted a lack of university resource support around Science Communication efforts or that the responsibility of identifying and integrating Science Communication resources fell on them. For faculty to find the needed equipment or resources for Science Communication, they described needing to identify equipment or software and the required training. One participant (1) noted,

It’s always on me to go out raise enough money to buy some piece of equipment. Some of that equipment can be fairly expensive, some of its fairly inexpensive and so you go out and buy this equipment and then I’ve got to figure out how to use it. I’ve got to figure out how I’m going to use it efficiently.

A few participants noted wanting more staff members to help with Science Communication efforts. Two different participants desired staff members who could do the Science Communication work on behalf of the faculty, while another requested staff who could be content creators for the faculty:

I could say ‘hey I have this idea for this’, and they can say ‘okay let’s schedule… three hours for us to come out and help you capture content. And then we’ll help you edit it down or we’ll edit it down and then you can communicate out the end product, and we’ll support that.’

(Participant 3)

Similarly, Participant 8 share,

There’s not a clearing house or a place where an organization can go to [Institution] and say ‘hey we’d really love somebody to come talk about home lawns or whatever’, and then that gets parceled down to somebody. That doesn’t seem to happen very often. And there’s really not an orchestrated way of doing it.

For these faculty, there was a desire for outsourcing Science Communication efforts to others who could create content that faculty could later share.

Other participants requested university level databases to help with Science Communication efforts. One (5) stated,

I can’t just go into our centralized database of materials and say ‘oh here’s a photo’ and it comes down with photo credit and all that kind of stuff so that when it comes to me, I can just put it into a presentation and it already has the credit there for who it’s from and like that that’s the kind of stuff that would be helpful.

Some participants thought shared equipment could be useful, though one (1) noted a possible negative outcome based on a lack of university support for Science Communication efforts:

What happens if we offer that and everybody at the Institute says ‘hey this is great idea’…I think that we don’t think through that initial piece of, ‘hey we have the suite of camera equipment, we have a couple of people that are dedicated to helping you capture content that know how to use this equipment and can use it really well, and you know take us up on it’. And then, if you have 30 faculty that say ‘Okay, I want to, I want to capture this content’, there’s two people there and they’re like ‘we can’t do that’. And so, then it ends up being either the favorite child or the hot topic of the moment, or whatever and it’s not available to everybody, it’s available to a handful of people.

Other participants described how the university structure may inhibit Science Communication efforts by creating silos within disciplines or even a disconnection between the university and the general public. Participant 10 and 2 stated:

Breaking down silos …. I reach out…I give guest lectures in electrical engineering I don’t have any background in engineering, the thing I’m working on is broadband and stuff and that’s applicable elsewhere. I think breaking those silos down and trying to reach out across campus and across departments, is important.” (Participant 10), “I tend to think a University College campus is a bit of a cocoon and unless you get off that campus and get out and actually see what’s going on it’s hard to stay in touch with what people are doing…because, again, the university itself is its own little microcosm.”

(Participant 2)

## Discussion & recommendations

This study provided insight into the unique public-facing science communication (Science Communication) challenges agriculture research faculty members face. The findings of this study provide a more in-depth understanding of faculty members’ unique experiences, as well as provide practical recommendations for communication professionals in various areas of work. Though prior research highlights numerous personal challenges faculty face when choosing to engage in Science Communication [14, 16], this study highlights some of the professional and institutional reasons that can make Science Communication difficult. For our participants, the challenges centered around the necessary knowledge, resources, and rewards associated with Science Communication work. For universities wanting to emphasize Science Communication going forward, this paper highlights some of the potential roadblocks.

We found faculty members in this study faced challenges across all three dimensions (personal, professional, and institutional) that hindered their engagement in Science Communication activities. At the personal level, time was a critical factor in choosing to engage in Science Communication. Though Poliakoff and Webb [25] defined time broadly (“I do not have enough spare time to participate in public engagement activities”), our participants went a step further to link time with the learning curve associated with Science Communication. Faculty members described how the learning curve associated with Science Communication was not worth allocating their time to in comparison to other activities. Previous work shows perceived lack of knowledge is a key barrier to participation in Science Communication [33]. This was also identified in our study. Specifically, faculty expressed a general disinterest in pursuing Science Communication knowledge on their own. Many participants described a need for Science Communication training opportunities to help develop specific science communication skills. To help research faculty engage in Science Communication, communications faculty members or other university communications professionals may consider offering workshops or other training opportunities that include step-by-step and time-efficient processes of engaging in Science Communication activities. Specifically, participants expressed interest in video production, mass-media interaction (interview techniques and developing stories with journalists), and social media training. However, faculty did express concern that media stories are often oversimplified or provided incorrect interpretations of their research findings. As such, it is important that communication support is an integrated process that ensures faculty oversight of content production.

The ability to effectively use Science Communication technologies was another area in which a knowledge deficit was communicated by faculty members in this study. To help combat this challenge, some participants mentioned having undergraduate or graduate students help with their communication or hiring communication specialists who are more familiar with social media norms and expectations. This finding may speak to an issue with some faculty feeling personally disconnected from Science Communication technology use or believing these skills should belong to a specific communication support role. Indeed, past research shows that tenured faculty feel less knowledgeable about science communication [16], and that science communication efforts are for specific support roles [14]. Based on these findings, we recommend training programs developed for faculty members include hands-on Science Communication technology training components such as camcorders and cameras, audio recorders, or editing software, especially if the intended trainees do not have the resources to hire communications support staff or students.

At the professional level, participants expressed difficulty in reaching out beyond their professional dsiciplines and connecting with public (non-scientific, student, or industry-based) audiences. Traditional graduate education does not focus on Science Communication [9, 10], but rather focuses on how to communicate within the discipline and academic community, which has a specific language and writing style dissimilar to Science Communication approaches. For some faculty, training in how to translate their work to a public audience could be useful and help break down some disciplinary norms around Science Communication. However, the deeper challenge of understanding how best to reach specific audiences may still be present. Faculty members’ struggles with disciplinary norms may also explain why many participants, other than those with Extension appointments, felt it was not their job to communicate their science to public audiences. Similarly, Parrella et al [16] found university scientists believed it was the job of the university to disseminate scientific knowledge, but they did not feel they were personally responsible for engaging with the public about their own science. The source of such disconnect between perceived institutional roles and values and personal responsibility is unclear. This furthers the importance of training that increases scientists’ positive attitudes and disciplinary association toward Science Communication. It also appears some disciplines or departments naturally lend themselves to Science Communication more easily than others. Specifically, departments or disciplines with higher percentages of extension faculty appointments will have more experience with public communication. As such, it is important for cross-discipline knowledge sharing such as partnering faculty from research-heavy disciplines with with extension appointment faculty in trainings and workshops.

Communication professionals, when designing training programs for faculty members, should carefully assess the professional makeup of their trainees. Based on our findings related to demographic characteristics like appointment type and tenure considerations, these factors can influence the learning needs and objectives of the participants. Prior research supports this observation [40, 41], suggesting that a one-size-fits-all approach may not be effective for all faculty members. Tailoring training programs to the unique needs and backgrounds of faculty can lead to more meaningful and impactful learning experiences. Based on our findings, including both extension and non-extension faculty in training sessions may provide valuable opportunities for knowledge sharing.

Lastly, at the institutional dimension, lack of resources within the department or university presented a challenge to engaging in Science Communication, especially when seeking out resources and training to utilize those resources rests on the efforts of the individual faculty member. Resources are crucial to engagement in Science Communication [33, 35]. To bolster the efficacy of Science Communication initiatives within institutions, it’s essential to consider strategic integration and resource allocation. Based on our findings, faculty members have highlighted the importance of readily available resources to effectively engage in Science Communication. One tangible solution, as proposed by participants, is the establishment of a central database that features media assets with proper accreditation. Such a database would provide faculty members with the tools they need to enhance their Science Communication projects. Furthermore, the inclusion of dedicated support staff to guide and assist in the implementation of these projects can further elevate their impact. However, the introduction of shared equipment or dedicated Science Communication personnel raises concerns about potential overwhelming demands. To navigate this, institutions should consider a collaborative approach in designing these resources. Engaging higher-ups and select faculty members in the planning and structuring process can ensure a system that caters to the needs of the faculty while maintaining operational efficiency. Such an inclusive design process is not only likely to yield better results but also foster a sense of collective ownership and commitment to the success of Science Communication within the institution. Prior research has shown that collaborative endeavors, especially those involving key stakeholders, often lead to more sustainable and effective outcomes [42]. Therefore, while our study offers initial insights, we recommend institutions delve deeper into the benefits of designing Science Communication resources in tandem with select stakeholders to ensure a comprehensive and user-centric approach.

While the challenge of resource availability is complex, particularly when considering institutional priorities, and heavily dependent on the university’s backing, the emphasis on research and teaching during faculty evaluations underscores a potential gap in institutional recognition for Science Communication and Extension efforts, especially for those without a formal public-facing appointment. Recognizing this challenge, it’s vital that universities consider integrating effective Science Communication efforts into their overarching strategic goals. For instance, a university might establish an annual ’Science Communication Research Award’ to honor research faculty who have made significant contributions to public-facing science communication. This not only promotes Science Communication endeavors but also provides a tangible benchmark for faculty to aim for. Furthermore, there is a need for the introduction of a diverse recognition system beyond the traditional Tenure & Promotion process. Universities could introduce grants or awards for Science Communication projects or even opportunities for faculty to present their efforts in university-wide showcases. Alongside these recognitions, offering workshops and training sessions to faculty members can serve dual purposes: equipping them to incorporate their science communication activities into their portfolios or dossiers, and providing a platform for them to receive recognition for their efforts. Additionally, establishing a feedback mechanism where faculty can share their insights and concerns can lead to more robust support and recognition for Science Communication initiatives, fostering a sense of institutional community and collaborative effort.
